# Clinician Experiences Assessing Work Disability Related to Mental Disorders

**DOI:** 10.1371/journal.pone.0119009

**Published:** 2015-03-19

**Authors:** Carolyn S. Dewa, Hiske Hees, Lucy Trojanowski, Aart H. Schene

**Affiliations:** 1 Centre for Research on Employment and Workplace Health, Centre for Addiction and Mental Health, Toronto, Ontario, Canada; 2 Department of Mood Disorders, Pro Persona, Wagnerlaan 2, The Netherlands; 3 Department of Psychiatry of Radboud umc, Nijmegen, The Netherlands; University of Perugia, ITALY

## Abstract

**Objective:**

Medical certification is one of the basic administrative mechanisms used by social policies aimed at income protection. The assessment of work disability is central to the income protection application. Yet, there is evidence suggesting that determining work disability related to mental disorders is challenging. Although essential to the disability application process, few studies have looked at physician and other clinician experiences with the process. However, this type of information is critical to developing processes to support providers who participate in the assessments. This purpose of this paper is to explore the experiences of physicians and other clinicians assessing public long-term work disability related to mental disorders.

**Methods:**

This is an exploratory and descriptive study using qualitative methods. Clinician input was gathered through focus groups and individual in-depth interviews. Verbatim transcripts were analyzed to identify recurrent and significant themes that arose during the focus groups and individual interviews.

**Results:**

Many of the experiences that the clinicians in this sample discussed related to the difficulty of trying to fill the roles of advocate and medical expert as well as the challenge of determining the impact of functional capacity and work ability. The findings also highlight the current gap in knowledge about the factors that affect successful functioning in general and at work in particular.

**Conclusions:**

Given the challenges created by the current state of knowledge, it may be useful to consider a category of “partial disability”. In addition, the fact that work disability depends on the interaction between the experience of the mental disorder and specific job requirements and the fact that people applying for public long-term disability are not working, it might be helpful to offer a clear description and guidelines of the meaning of work ability.

## Introduction

There has been increasing attention to the cost of work disability related to mental disorders. The Organisation for Economic Co-operation and Development (OECD) reports that a third to a half of new disability benefits claims are for mental disorders [1]. At the same time, there seems to be a perception that determining work disability related to mental disorders is challenging [2, 3]. Part of the difficulty is related to trying to understand the relationship between symptoms and work ability for people experiencing these disorders [4]. Furthermore, a common definition of work disability does not exist [5]. Rather, the definition is related to the disability benefit plan [6].

Perhaps, because a consistent definition of work disability does not exist and because there are standard criteria for medical diagnoses, criteria for work disability include (but are not necessarily limited to) information about medical diagnoses. Schienrenbeck [7] notes that medical certification is one of the basic administrative mechanisms used by social policies aimed at income protection. Consequently, in most OECD countries, regardless of the disability benefit process implemented, physicians comprise one of the key sets of players in determining work disability [5]. Wahlstrom and Alexanderson [8] describe the physician role as consisting of three components: (1) establishing a diagnosis, (2) assessing decreases in functional ability related to the diagnosed disorder and (3) assessing the relationship between functional ability and work ability. Depending on the system, parts of this role may be shared with other health providers who also are involved in the assessment process.

Although essential to the work disability process, in their review of the literature, Wahlstrom and Alexanderson [8] found few studies looking at physician experiences. Most of the studies were from the Scandinavian countries and only one was from North America. They also noted that there is a gap in the literature regarding how physicians [8] or other clinicians (e.g., occupational therapists, psychologists, social workers) perform these duties when addressing public long-term disability benefits. Yet, as they point out, this type of information is essential to develop processes to support providers who participate in the assessments.

The purpose of this study was to contribute to the sparse knowledge base regarding the experiences of physicians and other clinicians assessing work disability related to mental disorders. Data were gathered through focus groups and individual in-depth interviews with health care providers, who complete the public long-term disability forms for the Ontario Disability Support Program (ODSP), a provincial public long-term disability benefits program in Canada’s most populous province.

### Background

When clinicians are asked to assess work disability, challenges arise from the fact that they are being asked to straddle two disparate worlds—that of social legislation and that of medical practice [9, 10]. That is, when conducting a disability assessment, clinicians are asked to be an agent of two entities who may have different and sometimes conflicting agendas and purposes. On the one hand, based on his/her profession training, the clinician is expected to help the client. On the other hand, s/he serves as a gatekeeper for the disability system [11], a role that is not explicitly part of clinical training.

Based on their review of the literature, Wahlstrom and Alexanderson [8] identified two recurring themes relating to the role physicians serve in the work disability assessment context. The two themes included: (1) the conflict of fulfilling the patient advocate versus medical expert role and (2) the challenge of distinguishing the difference between functional ability and work ability. Because allied health providers are also trained to be clinicians, they can be involved in the assessment process. Thus, they also can be exposed to similar conflicts and challenges.

**Role conflict**. As patient advocate, the clinician seeks to help the patient as much as possible but as a medical expert for the disability benefit program, s/he is obligated to work within the constraints established by the plan. Gold and Shuman [6] describe the contrast as being one in which on the one hand, the clinician role is to establish a treatment alliance through empathy and non-judgmental listening. On the other hand, as the disability program’s medical expert, the clinician must interact with the same patient with objectivity, balance and a degree of skepticism.

**Assessing work disability**. In their study of sickness absence certificates in Sweden, Soderberg and Alexanderson [10] found that about 52% of the forms that they reviewed had missing information related to functional capacity. One interpretation of these results is that they may reflect the difficulty of deciphering the relationship between functional capacity and work ability. Zola [12] suggests that part of the difficulty may also be related to the fact that the symptoms of chronic conditions are not necessarily static. There is evidence that it is difficult to determine an absolute level of functional ability as well as the prognosis for the length of work disability [3, 13].

### Study Context

This study examines clinician experiences related to conducting the medical assessments related to mental disorders for the ODSP. ODSP is a provincial public long-term disability program of last resort that provides income support to Ontarians who demonstrate financial need and who have met the ODSP Act’s legislated disability assessment criteria. Assessment criteria include: a) having a substantial physical or mental impairment, b) an expected duration of the impairment for at least 12 months, c) the person’s activities of daily living in one or more of the following areas: personal care (e.g., bathing, grooming, dressing), activities in the community (e.g., banking, shopping), and activities in the workplace (e.g., being able to follow instructions) are substantially limited by the impairment. Finally, to be eligible for ODSP, a physician has to confirm the person’s diagnosis and either a physician or allied healthcare provider can assess the person’s impairment and functional limitations. The medical assessment is used in the eligibility determination for this public long-term disability benefit. Clinicians are asked to identify impairments associated with the presence of medical diagnoses. Thus, ODSP is based on a medical model of disability such that diagnosis and disability are intertwined [14]. However, the ultimate qualification decision is made centrally by ODSP program staff based on the assessments.

## Methods

An exploratory and descriptive study was conducted. This study used qualitative methods to gain in-depth information on clinician experiences. The research was approved by the research ethics board at the Centre for Addiction and Mental Health; all interview and focus group participants provided written informed consent to participate and for interviews and focus groups to be audio recorded.

### Participant Recruitment

Clinician input was gathered through focus groups consisting of 2–3 participants and individual in-depth interviews. The aim was for all participants to take part in a focus group. However, due to the conflicting schedules of these health professionals, it was not always possible for them to attend a focus group. In these cases, individual interviews were conducted to accommodate their schedules. Each participant was interviewed only once—either in a focus group or individual interview.

A purposeful sampling approach (i.e., participants were intentionally recruited based on their experience of completing ODSP forms) was used [15]. A snowball sampling strategy was also used to identify potential participants. Potential participants were approached by e-mail or phone and invited to take part in the study.

Interviews and focus groups were conducted in April-May 2013 by the first author and lasted an average of approximately 45 minutes. Data collection continued until saturation was achieved. Saturation was determined when no new information was being drawn from the sample; this was confirmed by two members of the research team (CSD and LT).

**Inclusion criteria**. There were three inclusion criteria. Since ODSP application forms can be completed by a diverse set of professionals, one of the criteria for inclusion was being a licensed provider in Ontario who was allowed to complete ODSP forms; this included general practitioners, psychiatrists, psychologists, social workers and occupational therapists. The second criterion was that the clinician had experience completing ODSP disability forms for people with mental disorders. The third criterion was that the person was currently practicing; the practice could be in either a specialized or general hospital or community agency in Ontario. Potential participants were required to meet all three criteria.

**Semi-structured interviews and focus groups**. The research question was informed by a review of the literature [16] and discussions with clinical experts on the assessment of work disability related to mental disorders. These experts included two family physicians. They were chosen because of their interest in public disability benefits and their experience completing these forms. They practice in an inner-city clinic that has patient income security as one of its missions. Their experiences with disability assessments helped to inform the development of the research question and interview guide.

The general focus of the focus groups and interviews was the diagnoses of mental disorders and the types of information that clinicians include when completing disability applications for their clients. A semi-structured interview guide was developed for use during the interviews and focus groups to ensure that subject areas and topics explored during data collection would be consistent [17]. Throughout the course of data collection, probes related to emergent themes were added to the interview guide.

Participants were asked to consider three primary questions: (1) What were the differences between the diagnostic information in the medical record and the disability application?, (2) What were their perceptions of the relationship between diagnoses and symptoms when assessing work disability? and (3) What were their experiences with completing the disability application?

**Description of participants**. A total of 13 clinicians participated in the study, with no participants dropping out or refusing consent. Two focus groups (comprised of n = 2 and n = 3 participants, respectively) and eight individual interviews were conducted; 9 participants were women and 4 were men. The participants represented the variety of clinical disciplines that are allowed to complete forms including social work (n = 1 participant), occupational therapy (n = 1 participant), psychology (n = 2 participants), primary care (n = 2 participants) and psychiatry (n = 7 participants). Based on their practice settings, they treated a broad range of patient populations. Of the 13 participants, 6 work with mood and anxiety disorders clinical populations, 5 with clinical populations with schizophrenia and 2 with clinical populations experiencing their first psychotic episode. The participants practiced in hospital-based (n = 5 participants) as well as community-based settings such as primary care (n = 2 participants), shelters (n = 1 participant), work disability speciality clinics (n = 3 participants) and community mental health programs (n = 2 participants).

**Analysis**. Focus groups and interviews were audio-recorded and transcribed verbatim. A constant comparative method of theme development was used [18]. Two members of the research team (CSD and LT) read all the transcripts and independently assigned codes to sections of text. Codes identified in and derived from the data were discussed by the two research team members until consensus was reached. Codes with similar characteristics were grouped together into categories. In turn, categories led to the development of overarching themes [19]. These themes are described in the results section below.

## Results

The categories that emerged from the responses to the semi-structured interviews and focus groups clustered into three primary themes. These themes included: the differences between the medical record and the disability application, the complexity of factors contributing to work disability and assessing work disability.

### Differences between the medical record and the disability application

There were a number of challenges identified with substantiating the diagnosis of a mental disorder. From the interviews, these included the dependence on self-report, reluctance on the part of clinicians to report secondary disorders and changing diagnoses over time.

**Lack of objective data**. The dependence on self-report and lack of objective data can make it challenging to validate diagnoses for mental disorders. Unlike physical disorders, there are no concrete tests with which a diagnosis can be confirmed. Participants also indicated that for some cases, the precise diagnosis cannot be determined. However, they agreed that in those cases, they are still able to determine whether a major mental disorder is involved (Table 1). It was also noted that over time and through gathering data, the certainty of a diagnosis can be improved.

**Table 1:** Theme of Differences between the Medical Record and the Disability Application Categories and Quotes.

Category	Quotes
Lack of objective data	… Also, I do see some people who are early in their psychosis, so in that case things [the diagnosis] may be a little less clear because you’re not sure whether it’s going to be bipolar disorder or schizophrenia, but it’s clearly a major mental disorder. (050913_001, p. 1)
	It’s really over a time and also gathering various data from various sources and so on, and also from our own treatment and dealing with the client that we are able to really increase our confidence whenever we are able to provide diagnoses. (042613_001, p. 2)
Secondary diagnoses	… I think their [ODSP] reason for doing the form is to determine whether they’re able to work or not. So, if the first diagnosis already makes them unable to work, then I feel that they may not necessarily require the second supporting diagnoses as well, and if they’re [secondary diagnoses] not needed, then maybe they shouldn’t be relayed to the government or to the workplace or wherever those forms are going. (050713_002, p. 2)
	… I think there’s a lot of reporting bias that comes with clinicians on disability forms. And, I say, yeah, there are probably two things, a tendency to minimize adherence issues, minimize personality factors, and minimize the role of substance use factors. (042613_001, p. 6)
	…sometimes unless somebody is stable it’s hard to distinguish whether it’s [the symptoms] are a function of the illness or whether indeed it is a personality disorder. (042413_001, p. 12)
	So, if you’ve done the rigour around the reports of symptoms, you’re at least confident about what may be primary, but then it gets more difficult the more diagnoses people have to be certain which one is impairing one and which one is not, or which symptoms from what diagnoses are leading to what impairment. (042613_001, p. 3)
	…So, a lot of my patients would have a Grade 2 to Grade 3 arithmetic skill. Well, that’s a huge disability in society, and it’s a huge disability for day-to-day living… So, they can read, they could spell, but in terms of day-to-day functioning, for a lot of them it’s terrible. And, they have huge social judgment problems that are just massive. (042913_001, p. 3)
	I’ve had people who can’t read, and no matter how often you send them off for literacy, they just can’t read, so it becomes an issue of safety in the workplace too, right? Because, if they can’t read WHMIS [Workplace Hazardous Materials Information System] information, for example, or they can’t read instructions from their employers, then I’d be concerned about their safety. (050913_001, p. 2)
	So, there would have to be … it would be a combination of comorbid diagnoses, so the more diagnoses someone has, the more likely you’re going to consider total disability as a result of their mental disorders, and the more psychosocial stressors in the absence of supports. (042613_001, p. 8)
Changing diagnoses	The other thing is that over time sometimes also the diagnosis can change, either because the person is better or sometimes other things do occur, so the diagnosis might also change. Or, certain things that were not transparent initially become transparent later on. So, sometimes when we do an assessment or a primary diagnosis, and sometimes maybe there could be something during secondary diagnosis but we’re not sure, but over time when we notice that the primary diagnosis is resolved, suddenly it’s really the secondary one that becomes the primary. Often, we see that in people that deal with depression and let’s say social anxiety and let’s say OCD. At the beginning it could really be depression, but once depression is resolved, suddenly we see this social anxiety that becomes a significant barrier. (042613_001, p. 3)

**Secondary diagnoses**. Although participants agreed that they were generally confident in the primary diagnoses (i.e., the disorder to which the disability is attributed) on the forms, there was more concern regarding the completeness of information provided about secondary diagnoses. Participants offered several reasons for not reporting these additional diagnoses. In some cases, secondary diagnoses were viewed as unnecessary to the application. That is, if symptoms related to the primary diagnosis were considered sufficiently severe such that it was perceived that a secondary diagnosis would not change decisions regarding the extent of work disability, other diagnoses were not included on the forms.

A second reason for omission of secondary diagnoses was attributed to stigma that is associated with some of these disorders, such as substance abuse and personality disorders. Although these disorders or the symptoms associated with these disorders are described in the medical record, the presence of these disorders may not be reported on the application forms.

Third, the clinicians reflected on the difficulty in diagnosing Axis II disorders, particularly in the presence of Axis I disorders. This may be another reason why clinicians could be hesitant to include Axis II disorders as secondary diagnoses on the forms.

Although they indicated that it could be difficult to diagnose an Axis II disorder, they also acknowledged that the Axis II diagnoses (i.e., personality disorders and intellectual disabilities) could also contribute to disability.

There also was agreement that comorbid disorders greatly increased the risk of having a disability. It appears that clinicians may be selectively reporting secondary disorders such that they report them if the severity of primary disorder is not considered sufficiently severe. But, there is a hesitancy to report them if the comorbid disorder such as in the case of Axis II disorders, could result in either stigmatization of the applicant or the clinician is unsure of the diagnosis.

**Changing diagnoses**. Finally, participants noted that sometimes there was a difference between the primary diagnosis for the onset of disability and the diagnosis responsible for the maintenance of the disability. This suggests that for someone who has a prolonged disability, the primary diagnosis causing the disability may change over time. The diagnosis identified as the original cause of the disability may not be the diagnosis that explains the continuing disability.

### Complexity of Factors Contributing to Work Disability

The participants agreed that there are a number of factors that contribute to work disability (Table 2). They not only include clinical diagnosis and symptoms, but also the level of functioning (e.g., psychological impairment and cognitive impairment), psychosocial problems, and for some participants, job characteristics. The following section highlights the discussion regarding these factors and how they are currently communicated.

**Table 2:** Theme of Complexity of Factors Contributing to Work Disability Categories and Quotes.

Category	Quotes
Clinical diagnosis	Diagnosis is just simply a label that they could communicate, and that’s what I’ll often tell my clients. It’s really to me the diagnosis means nothing. Definitely symptoms and definitely functional status [are important to determining disability]. (050713_001, p. 5)
	So, they may be able to do it sometimes, right, but it’s the consistency that they’re able to attend. No one is going to employ you if you’re missing 50% of the time. You may be good operating at 75%, 50% of the time. You’re not generally employable because nobody’s sick leave is going to support that. (042613_001, pp. 15–16)
	…you have intermittent illnesses but they may be recurring within a frequency that the person’s never going to be able to work. So if they’re ill for a month and well for a month there’s no way they’re going to be able to sustain a job and yet they’re not ill all the time. (042413_001, pp. 12–13)
	If I’m saying a person is depressed and what are their impairments, well, they have difficulty with their motivation, they have difficulty being positive about life, sometimes it’s hard to get out of bed, these things don’t sound as stark and severe as they do in the psychotic illness. So, with those ones especially, I try to provide hospital discharge summaries or any sort of evidence that I can that an impairment is severe enough to prevent somebody from working. (042613_002, p. 2)
	…However, I think it’s more difficult than with physical illnesses to show it [a mood disorder] disables them. If somebody has a broken leg. it’s going to be obvious that they can only walk with crutches or ride in a wheelchair. Whereas, if they have a symptom like fatigue or lack of motivation. it’s much harder to document the specific way it makes them disabled. (042413_001, pp. 3–4)
Functional capacity	I don’t know about diagnosis, I feel like it’s a level of function versus diagnosis… So, it is a level of function, because the same diagnosis can affect different people differently. Some people are more resilient than others, so it is important for me to specify on the application form that the level of function is very low, particularly when it comes to work, paid employment. (042313_001, p. 5)
	But, with respect to psychosis and everything, I think it’s very hard because there are certainly some people, I certainly have some patients who have still some symptoms, but I mean my best patient has never been, never for a day, on ODSP, works, pays taxes, has more investments than I do, and is a fabulous citizen. He’s not going to set the world on fire socially, but he’s really, really, really good, and stays on his medication, but he still has symptoms. I mean if you ask him are there Zoroastrians that he still sees, he’ll say yes. (042913_001, p. 11)
	The other part I think that’s the trickiest part is this idea of equating symptoms to functional impairment. It’s not always clearly defined, and I think there’s a way to structure and one of the things I often present or educate people about, for example, how problems with mood regulation impact interpersonal functioning. How problems with cognitive functioning impact ability to carry out tasks consistently. How energy deficits and energy and motivation impact people to stick with a job or reliably attend to duties. (042613_001, pp. 15–16)
	If somebody is scoring really slow in processing speed, chances are they wouldn’t be able to perform at a regular pace. If somebody’s having difficulty with their executive function, so inability to plan and get organized, they’re likely to have to struggle a lot, people who have difficulty with attention and working memory. (042313_001, p. 2)
	So, just an example, you may get a client that’s quite stable and they have bipolar disorder, but as soon as they’re entering a work environment they’re unable to cope, even though from a symptom perspective they may be stable. But there is an underlying disability. (042413_001, p. 5)
	…But, there are often other issues like frustration and inability to cope with changing environments that can have a significant impact. (050913_001, p. 2)
Psychosocial factors	So, that’s where Axis IV [psychosocial and environmental problems] really is a consideration I think in someone’s ability to function and someone’s ability to recover or the likelihood it’s going to be permanent. (042613_001, p. 8)
	…I have two clients who came to me when they were 16. They were identical twins. One is now in our alumni program and he sees us once every six months. He’s moving on with his life, really does not need anything else. The other one is now on ODSP, supported housing here in xxx, and we’ve just referred him to ACT [Assertive Community Treatment] because he needs weekly check-ins. So, again, identical twins you’d think that they … I also agree with the amount of support someone has and the amount of family burn out. The family was done by the time the second twin got sick. They’re a lovely family, but they just couldn’t do what they did for the first twin. So, without a tremendous amount of support I think in the early stages and then throughout, it’s like a marathon for these families, so it’s hard for them to keep it up. (050713_001, p. 6).
Role of job characteristics	I think we really need to start with a hypothesis, which is, a person is employable unless shown or proven otherwise. (042613_001, p. 13)
	… I picture two realms. When I picture realms at work, I picture the lowest common denominator of employment, which would be I picture the parking lot attendant. And, then I picture the best job that they’ve ever had, which is their ability to function at their best level. It’s easier when people have a work history or not, and said is there something they can do within that range. And, if they can do something in that range, they’re not totally disabled. (042613_002, p. 12–13)
	Or, if you have a lot of problems like my patients do, it isn’t even so much their actual ability, it’s that they can’t do anything under time pressure, and a lot of jobs now are under a lot of time pressure. They would be fine if they could plod along at their own pace, they’re excellent workers. They are excellent workers. Routine, something totally structured that they can cope with for a few hours a day, they would be great. They would be the employee you would want to have because they would be on time, dedicated and every day functioning, and not be bothered by the routine because they love the routine and the structure. But, a lot of jobs aren’t like that. (042913_001, pp. 8–9)
	That’s interesting, because with private insurance you always have the option of looking for accommodation. So if the person could go back to work in a quiet room or something, the insurance company and the employer can negotiate and figure that out. But I never really think of that so much as an option with something like ODSP, because there really is no relationship between the provincial funder and the employer where you could negotiate conditions and accommodations for returning to work… (042413_001, pp. 6–7)
	It’s easier if a person actually is on a leave from work and you get a clear sense of what their work environment and demands are, that may be an easier situation to think of accommodations. As opposed to somebody starting from scratch, being unemployed and possibly never have worked before. (042413_001, p. 7)

**Clinical diagnosis**. There was overall agreement that for most cases, work disability cannot be based on the primary diagnosis alone. Rather, the diagnosis was regarded as an indicator of a vulnerability to work disability. The diagnosis seemed to be viewed as short-hand that was used to identify the constellation of symptoms that a person experienced. One participant described the diagnosis as a label.

Participants also indicated that in their disability assessment they considered the intensity with which symptoms related to mental disorders recur and interfere with ability to consistently work. The interviewees also spoke about the difficulty in communicating how some of the symptoms of depression could lead to work disability.

There seemed to be consensus that meeting diagnostic criteria for a mood disorder did not adequately communicate the level of impairment. Indeed, all participants indicated that although not specifically requested, they felt compelled to provide supplemental information for this purpose, to adequately communicate the level of severity. Examples of the supplemental information included medical records, history of hospitalization and emergency department use as well as test scores where available.

**Functional capacity**. When determining work disability in mental disorders, participants suggested that the level of function was of greater importance than symptoms alone. Thus, people with the same level of symptoms may be differentially affected in their functional capacity.

In addition to the symptoms, there also seems to be a question of how an individual deals with the symptoms. The examples given indicate that symptoms such as delusions are not disabling for everyone; the key to successful employment is the ability to cope with the symptoms.

Participants consistently identified impaired cognitive and psychological functioning as contributing to work disability. With regard to cognitive function, one of the participants pointed out that there are several dimensions of cognitive functioning. Among the different dimensions, she identified the importance of processing speed, executive function and working memory.

Respondents also referred to the importance of psychological functioning. Although a critical component of disability, they noted that often, the disability application forms do not specifically ask for information about psychological functioning, such as the patients’ abilities to cope with the daily demands of the work environment.

**Psychosocial factors**. There was agreement that psychosocial and environmental problems, such as lack of housing and social support, are related to the level of work disability. In addition, when these factors are lacking, they may hinder recovery. One participant offered the example of twins who both experienced psychosis but had different outcomes. She attributed part of the difference—the success of one and the continual struggle of the other, to the different levels of support received by the two twins.

**Role of job characteristics**. Many ODSP applicants are not in the labour force. Yet, when assessing work disability, it becomes necessary to consider jobs and job characteristics. Participants seemed to be divided into two broad groups based on their perspectives on the role of job characteristics in determining work disability. In one approach, participants started with the assumption that a job does exist and the question is solely whether someone is able to function. The focus was on the individual. Thus, the question of work disability assumed there was a suitable full-time job in the competitive job market; the question was whether the applicant could do it.

Alternatively, another group of participants described an approach in which the characteristics of a potential job were considered. This difference in philosophy may result in a different approach for determining work disability. For the latter group, the absence of a job context makes it particularly challenging to determine work disability. A contrast was drawn between a return-to-work situation in which an employer could offer work accommodation versus an unknown employer who has no relationship with a potential employee who needed accommodations before starting work.

### Assessing Work Disability

During the disability assessment process, clinicians are asked to fill two roles that can at times seem to be competing (Table 3). One role is that of a patient advocate and clinician. In this role, the goal is to help patients achieve optimal health. The second role is that of an assessor for the disability benefit insurer. In this role, the goal is to report whether an applicant meets the ODSP’s disability criteria. There seems to be a tension inherent in simultaneously trying to meet the requirements of being a patient advocate and an unbiased assessor.

**Table 3:** Theme of Assessing Work Disability Categories and Quotes.

Category	Quotes
Clinician role	In fact, that’s why I often don’t fill out ODSP. I don’t want to give my client the message, which I think ODSP gives, that I don’t think you can ever work again in a meaningful employment. And, that’s where I weed out 50%. (050713_001, p. 10)
	I think work is good…The problem is I do know that some people at some points are clearly not able to work and need some financial support. That doesn’t mean that as they progress, often the symptoms can change over time and particularly if they get therapy. But, you know, it’s often a longer process, particularly with people with schizophrenia for example. And, so I’ve often had people who are on ODSP, who wanted to get off ODSP because they wanted a meaningful role… But, I think part of the issue with the ODSP is there are some folks who think of ODSP as that’s their life work. If they’re on ODSP, they’ll always be on ODSP, and I don’t think that’s necessarily a good way of thinking of it. (050913_001, pp. 2–3)
	Personally, I think there’s a huge issue… we’re advocating for our own patients so we’re kind of biased to try to help them get what they’re looking for. It might be fairer for people to have these forms filled out by an independent person. But that would be a lot more expensive too probably to be paying people to do that. (042413_001, pp. 17–18)
	…If somebody is coming to me with a substance or depression problem and they want to be on disability, I always counsel them that it’s much more difficult, and I find that I have a much higher sort of challenge rate or appeal rate with that group. And, I always also counsel people that I’m going to put things in the most stark terms possible to explain it, rather than the way I might talk to them in person, which is to say, in person I might be saying, well, you have these disabilities, but look how well you’re doing in this and that regard. On the form, I’m going to be talking about the disability and its extent. I always tell people that I will never lie, but I’m going to have to be quite stark about it to make the argument. (042613_002, pp. 1–2).
	I think that also as much as we can when we complete the forms we try to do what is within our profession. The way I put it in my mind, is whenever I complete those disability forms, hopefully I try my best to be as genuine and impartial as possible. So, I really put whatever I believe in regards to the person, but I always say to the person whether it’s going to work out or not, it’s just not within my control, and this is not my area. So, I’m doing my job to resolve what I’m being asked to do, but in terms of the outcome, what’s decided, it is actually not within my control. (042613_001,p.9)
Partial disability	They might be able to work part-time. It may be an issue around not total disability. It’s just partial disability. (042613_001, p. 9)
	You have to make a more clear-cut decision, there’s no grey area, they either are disabled or they aren’t. I’ve never really thought of a way to say they’re partially disabled and can partially work. (042414_002, p. 7).

**Clinician role**. Part of the challenge may be related to the clinician role. Because of the possibility of improvement, participants described a hesitation to make absolute statements regarding disability and resisted communicating such a bleak message to their patients. Participants recognized a potential for change over time. In addition, their concerns seemed to suggest a role for hope in the treatment process. They view the ability to work as fluid.

On the other hand, the participants also view themselves as conducting the assessments on behalf of the disability benefit program. This obligates them to provide as accurate information as possible. They are being asked to set aside their advocacy roles. Yet, they do not make the final determination of work disability.

**Partial disability**. Participants suggested that one of the ways in which the assessment might be made easier would be by introducing the concept of partial disability. They wanted to be able to assess disability along a continuum of ability rather than in absolutes.

## Discussion

This paper is one of the few Canadian studies that have explored the experiences of clinicians assessing public long-term disability related to mental disorders. As reported in the literature from other jurisdictions, many of the experiences that the clinicians in this sample discussed related to the difficulty of trying to simultaneously fill the roles of advocate and medical expert as well as the challenge of determining the impact of functional capacity and work ability.

### Clinician Advocacy

One of the ways that the ODSP process seems to have reduced the conflict of the two roles is by leaving the final qualification decision with an adjudicator. In the process, the clinician’s role is to provide an assessment. Yet, the clinicians still acknowledged that they felt responsible to fill an advocacy role. They seemed to address the conflict of the advocacy and expert roles in a number of ways.

In an effort to advocate for their clients, they provided as much supplemental information (i.e., medical records, history of hospitalization and emergency department use) as available to assist the adjudicator and to support the application. There was anxiety that the symptoms and impairments related to mental disorders would be trivialized. However, because the inclusion of supplement material is left to the discretion of the clinician, this can lead to variability in the type and amount of information provided. A potential consequence is perceived inconsistencies in qualification decisions.

At the same time, there was an inclination toward not reporting diagnoses. This was attributed to the perception that particular diagnoses (i.e., substance abuse and personality disorder) could negatively label a person with the insurer. The decision to omit the report of multiple diagnoses also was made when it was perceived unnecessary. Furthermore, as client advocates, participants also expressed a need to “protect” clients who could be stigmatized by benefit receipt from applying for the public long-term disability benefit.

As experts, the clinicians also seemed careful to only provide information of which they were certain. Thus, diagnoses for which they were not sure were not reported. There was also a concern that because the course of mental disorders is not static [6], it is difficult to anticipate the length of the period between severe episodes. If the periods are short, it may be difficult to maintain employment.

### The Medical Model of Disability and DSM

Few studies have described the struggle that clinicians face when assessing disability related to a mental disorder. Our results highlight the difficulty inherent in a medical model of disability. In a medical model, symptoms and functioning are considered together. Yet, there are factors other than diagnoses that could contribute to disability. This is one criticism of the DSM-IV (the North American standard for the classification of mental disorders until 2013); it reflected a medical model of disability. Consequently, it did not distinguish symptom criteria for a diagnosis from criteria to determine clinical significance [20]. DSM-IV required someone to demonstrate a level of distress or disability to fit the criteria for a diagnosis [20]. As a result, it was difficult to discuss disability apart from a diagnosis. Under a medical model, the diagnosis is a key factor and a major point of preoccupation in determining disability. Yet, the diagnosis does not always seem to be the key determinant of disability. This adds to the challenge faced by the clinicians who are conducting the assessments.

In contrast, in a social model of disability, the assessment of a diagnosis is separated from the assessment of disability. For example, the World Health Organization (WHO) distinguishes between disease and function with the International Classification of Diseases (ICD) and International Classification of Function (ICF) [21]; one classification is focused solely on disease and the other on function. In addition, a social model recognizes that disability also could result from contextual factors [22]. In this way, a social model of disability acknowledges the contribution of non-medical factors that a medical model of disability does not. It also gives weight to the context and accessibility of resources in assessing disability. In turn, a social model creates incentives for funders to increase the accessibility of resources that would diminish the effects of disorders.

The DSM-5 (the North American standard for the classification of mental disorders introduced in 2013) moves closer to a social model of disability by separating diagnosis and functioning. The DSM-IV had a separate Axis (Axis V) for the assessment of functioning. In contrast, the DSM-5 requires functioning to be measured separately. It suggests the use of the WHO Disability Assessment Schedule, Version 2.0 (WHODAS 2.0) [23] which was developed based on the ICF. However, Konecky et al. [14] and Gold [21] point out that the WHODAS 2.0 does not completely address the gap created with the removal of Axis V. They assert that part of the difficulty is related to the fact that the DSM-5 also identifies the WHODAS 2.0 as a “measure for further study” [14, 21]. In addition, the WHODAS 2.0 defines problem in functioning as “difficulties due to health/mental health conditions.” [23] (p. 747). That is, the problem in functioning may be related to the presence of a health/mental health condition.

Moreover, the DSM-5 also indicates, “Mental disorders are usually associated with significant distress or disability, social, occupational, or other important activities.” [23] (p. 20). This has also become a focus for debate. On the one hand, it can be interpreted as the DSM-V indicates that dysfunction is not an obligatory characteristic of a mental disorder. On the other hand, critics point out that it has not gone far enough to separate the two because “…a model of illness that includes impairment as a threshold criterion in its definition of psychiatric disorders does not map easily onto a model that separates disorder and related impairment and disability." [21] (p. 176) [14]. In either case, the DSM-5 is a marked move away from a medical model of disability; it is the magnitude of the move that is in contention.

In the future, it will be important to see how changes in the DSM-5 affect disability assessment and clinician experiences in North America. Will the assessment of disability become less challenging? With the DSM-5 elimination of the multi-axial diagnosis, some of the challenges that the clinicians reported were related to a reluctance to report Axis II diagnoses are eliminated. Furthermore, the potential separation between diagnosis and disability may also remove the need to report potentially stigmatizing comorbiditities in assessments. However, Narrow et al. [24] also call attention to the fact even in the ICD-10, there are some disorders such as personality disorders, substance dependence, and conduct disorders that have activity limitations as part of their symptoms criteria. Thus, the separation between the assessment of diagnosis and disability for some disorders may continue to be inter-related.

Consistent with previous research (e.g. [4, 13, 25]) and the ICF, we found that clinicians describe the assessment of work disability related to mental disorders as being complex and entailing both medical and non-medical components. However, there seemed to be the perception that the public disability forms may not adequately allow for the communication of this complexity. Despite the fact that the forms distinguish between the presence of a disorder, the impairment resulting from the disorder, and the way in which these impairments affect participation, the main focus of these forms is on the clinical diagnosis and severity of symptoms (across several domains, e.g., mood, impulse control). Typically, public disability forms do not systematically ask for information regarding cognitive functioning, psychological functioning, intellectual capabilities and psychosocial and environmental problems. However, from a clinical perspective, these factors should be taken into account when assessing work disability for people with mental disorders. This perspective is supported by the literature, showing that work disability related to mental disorders is multi-factorial and related to a number of psychosocial and environmental factors (i.e., coping, social support; [26, 27]).

With the DSM-5’s disassociation of diagnosis and disability, disability assessment may become more straightforward. However, it should be noted that one of the limitations of the WHODAS 2.0 is related to the fact that it does not assess environmental and personal factors [14, 28]. To the extent that clinicians use these factors to determine disability, assessments may continue to be challenging. It will also be important to examine how assessment is affected when the DSM-5 is used in a disability system structured around a medical model.

### The Role of Job Characteristics in Assessing Work Disability

Perhaps, it is also because of the desire to meet the expectation as identified experts, that there also seemed to be discomfort in determining work disability. Gold and Shuman [6] assert that the “most significant factor in the assessment of the effect of any psychiatric disorder on work function is the interaction of the specific impairment with specific job requirements.” (p. 73). The results of this study emphasize the relationship between determining work ability and the need to understand the job requirements. Our study is one of the few to observe the dilemma faced by assessors. Because public long-term disability applicants are not in the labour force, they do not have jobs. Thus, clinicians are not supplied with specific job requirements and are left to their own judgments. The differences among the work disability criteria used by insurers may add to the confusion.

There are at least two approaches that could further reduce clinician discomfort. One is to create a process and infrastructure that requires independent assessments by clinicians trained in occupational health assessment. For example, in jurisdictions such as the Netherlands, there is a multi-clinician approach to assessing disability that includes an occupational health physician and insurance physician who are separate from the treating physician and a reintegration specialist.

A second approach is to develop tools and training for clinicians. The latter could be viable for a system that does not have the resources to establish an infrastructure and processes for independent assessments. In this case, it would be important to provide clarity regarding job requirements. For instance, what are the levels of physical, cognitive, affective and social work demands that a person should meet to be considered to have work ability [6]? Guidelines and education about how to assess each of these domains especially in circumstances where there is no specific job to consider, could lead to better common understanding and consistency in assessment.

### Strengths and Limitations

This study is one of the few to describe the work disability assessment process for mental disorders from the clinician’s perspective. However, the present findings should be interpreted in light of the study’s limitations. The experiences of the participants may not necessarily be representative of all clinicians. However, despite differences in the organization of social security systems and jurisdictional contexts, similar themes have been identified in other studies and literature reviews [8, 11, 29], supporting the validity of the present findings. Nevertheless, the information gathered from this study will contribute to a better understanding of the experiences of clinicians assessing people for work disability benefits. In addition, the findings from this study point to areas of inquiry for future studies.

In addition, two interview modes were used. This could have resulted in a difference in how participants responded or in what they focused on in the discussion. Unlike an individual interview in which the interaction is with the researcher, focus groups create a setting in which there is the potential for more interactions. In focus groups, participants can interact with one another and the role of the researcher is decentered [30]. However, because focus groups bring more perspectives together and there is social interaction among multiple participants, they will not elicit as in-depth a response as a one-to-one interview. Thus, there is a trade-off between exclusively conducting one-on-one interviews that could lead to a more detailed set of data versus the use of focus groups that offer breadth over depth.

## Conclusions

The experiences of these clinicians highlight important considerations for public disability benefit programs. Even in a system in which clinicians do not make the final decision about qualifying for ODSP, they experience conflict emerging from the two roles of advocate and medical expert.

These findings also highlight the factors contributing to the difficulty in determining long-term disability related to a mental disorder. Among them is the current gap in understanding about the factors that affect successful functioning in general and at work in particular. Without this knowledge, the fluidity of the symptoms of mental disorders and the absence of a job, makes it nigh impossible to offer a prognosis about future work functioning.

Given the challenges created by the current state of knowledge, it may be useful to consider a category of “partial disability”. This category addresses the difficulty in offering a prognosis and also could address potential stigmatization of receiving a benefit that otherwise would signal complete and potentially permanent disability. It also would allow the hope of working to be fostered. In addition, the fact that work disability depends on the interaction between the experience of the mental disorder and specific job requirements and the fact that people applying for public long-term disability are not working, it might be helpful to offer a clear description and guidelines of what work ability means in terms of physical, cognitive, affective and social work demands.
